# Natural and anthropogenic radioactivity levels and the associated radiation hazard in the soil of Oodalia Tea Estate in the hilly region of Fatickchari in Chittagong, Bangladesh

**DOI:** 10.1093/jrr/rru054

**Published:** 2014-07-29

**Authors:** Nurul Absar, M. Mashiur Rahman, Masud Kamal, Naziba Siddique, Mantazul Islam Chowdhury

**Affiliations:** 1 Department of Computer Science and Engineering, BGC Trust University Bangladesh, Chandanaish, Chittagong, Bangladesh; 2 Department of Physics, University of Chittagong, Bangladesh; 3 Radioactivity Testing and Monitoring Laboratory, Bangladesh Atomic Energy Commission, Chittagong, Bangladesh

**Keywords:** radioactivity, soil, high-purity germanium detector, radiation hazard, dose rate

## Abstract

Radioactivity in the soil of a tea garden in the Fatickchari area in Chittagong, Bangladesh, was measured using a high-resolution HPGe detector. The soil samples were collected from depths of up to 20 cm beneath the soil surface. The activity concentrations of naturally occurring ^238^U and ^232^Th were observed to be in the range of 27 ± 7 to 53 ± 8 Bq kg^−1^ and 36 ± 11 to 72 ± 11 Bq kg^−1^, respectively. The activity concentration of ^40^K ranged from 201 ± 78 to 672 ± 81 Bq kg^−1^, and the highest activity of fallout ^137^Cs observed was 10 ± 1 Bq kg^−1^. The average activity concentration observed for ^238^U was 39 ± 8 Bq kg^−1^, for ^232^Th was 57 ± 11 Bq kg^−1^, for ^40^K was 384 ± 79 Bq kg^−1^ and for ^137^Cs was 5 ± 0.5 Bq kg^−1^. The radiological hazard parameters (representative level index, radium equivalent activity, outdoor and indoor dose rates, outdoor and indoor annual effective dose equivalents, and radiation hazard index) were calculated from the radioactivity in the soil.

## INTRODUCTION

In Fatickchari, in the northeast area of the Chittagong district of Bangladesh, there is a big tea garden named Oodalia Tea Estate at latitude 22°36′39″–22°39′41″N and longitude 91°45′6″–91°51′15″E. Tea is one of the main economic crops in Bangladesh. Many people are directly involved in the tea processing for this garden, which is ∼3000 km^2^ in size, and they may not be aware of the effects of radiation contamination on health. So, it is important to study the distribution of the various radionuclides present in the soil of the tea garden and to make an assessment of the radiation exposure resulting from these terrestrial radionuclides. The radioactivity of soil samples collected from depths of up to 20 cm beneath the soil surface of the tea garden was measured using a high-resolution HPGe detector. The radioactive constituents of soil, and in particular the natural radionuclides ^238^U, ^232^Th and ^40^K, are one of the main sources of the external γ-ray exposure to which people are regularly exposed naturally [1]. The study of radionuclides in the soil is a fundamental step in understanding the behavior of radioactivity in an ecosystem; these natural radionuclides emit radiation as they disintegrate, and this radiation contributes to the total absorbed dose for people (via ingestion, inhalation and external radiation) [2]. It is also important to assess any artificial radioactive contamination due to fallout. The objective of this study was to determine the activity concentration of natural and artificial radionuclides in the soil of the Oodalia Tea Estate and from this to derive the radiation hazard parameters so as to establish the radiation background database. The present work provides background data on the natural and artificial radioactive isotopes present in a typical tea garden in the hilly area of Chittagong. This is of great importance for the development of radiation protection and also for the assessment of uptake of these radionuclides from the soil into tealeaves and thus into the food chain, and in particular into the human body.

## MATERIALS AND METHODS

### Collection and preparation of samples

Soil samples were collected at depths of 0–5 cm, 6–12 cm and 13–20 cm from five hilly sites at the Oodalia Tea Estate in the Fatickchari region of Chittagong district, Bangladesh. The sites were: (i) Horindra Leaf Park (HLP) Section 17, near the Bangladesh Tea Research Institute (BTRI) in the south line region, (ii) HLP Section 5, near BTRI in the south line region, (iii) North Line Region (NLR) Section 1, (iv) Kala Phani Region Section 6B, and (v) NLR Section 11/3 of the Tea Estate. The geographical locations are listed in Table 1. All the samples were collected from June to July 2011.

**Table 1.** Sampling sites and their geographical positions

Location	Sampling location	Depth cm	Sample code	Geographical position	
				Latitude	Longitude
1	Horindra Leaf Park 1, Section 17	0–5	HLP1-01	22°33′55.96″N	91°43′55.89″E
		6–12	HLP1-02	22°33′55.96"N	91°43′55.89"E
		13–20	HLP1-03	22°33′55.96"N	91°43′55.89"E
2	Horindra Leaf Park 2, Section 5	0–5	HLP2-04	22°37′59.90"N	91°44′36.05"E
		6–12	HLP2-05	22°37′59.90"N	91°44′36.05"E
		13–20	HLP2-06	22°37′59.90"N	91°44′36.05"E
3	North Line Region, Section 1	0–5	NLR1-07	22°36′39.78"N	91°51′15.79"E
		6–12	NLR1-08	22°36′39.78"N	91°51′15.79"E
		13–20	NLR1-09	22°36′39.78"N	91°51′15.79"E
4	North Line Region, Section 11/3	0–5	NLR2-10	22°34′28.14"N	91°49′1.71"E
		6–12	NLR2-11	22°34′28.14"N	91°49′1.71"E
		13–20	NLR2-12	22°34′28.14"N	91°49′1.71"E
5	Kala Phani Region	0–5	KPR-13	22°39′41.22"N	91°45′6.95"E
		6–12	KPR-14	22°39′41.22"N	91°45′6.95"E
		13–20	KPR-15	22°39′41.22"N	91°45′6.95"E

Soils were collected using a soil corer. After removing stones, grass and any other biological materials, the samples were crushed, sieved through 2-mm mesh, homogenized, dried in an oven at 120°C for 3 d, pulverized, weighed, packed into cylindrical screw-cap plastic containers of 6.5 × 7.5 cm, then sealed and stored for 4 weeks to establish secular equilibrium between the ^238^U and ^232^Th series with their daughter radionuclides [3, 4].

### Measurement of radionuclides

The activity concentrations of radionuclides were measured using γ-spectrometry with a p-type coaxial high-purity germanium (HPGe) detector having a germanium cylinder crystal with 52 mm outer diameter and 49.5 mm height, a relative efficiency of 20% and a resolution (FWHM) of 1.80 keV for the 1332.5-keV γ-ray energy of ^60^Co. The detector was coupled to a 8192-channel computer analyzer and shielded using a cylindrical 5.08-cm-thick lead shield with fixed bottom and moving cover. The full energy peak efficiency of the radionuclides was measured using IAEA reference samples RGU1, RGTh1 and RGK1 [5]. The background distribution resulting from the naturally occurring radionuclides in the environment around the detector was determined by measuring the activity concentrations of an empty plastic container in the same manner as the samples. The background was subtracted in order to get net counts for the samples. The counting period for all the samples was 20 ks. The results were expressed with the confidence limit of ± 1σ. The activity concentrations of ^238^U were assessed from the γ-ray lines of the daughter radionuclides ^214^Pb and ^214^Bi; the energy regions selected were 295.2 and 351.9 keV for ^214^Pb and 609.3, 1120.3 and 1764.5 keV for ^214^Bi. The activity concentration of ^232^Th was determined from the 583.19-keV γ-ray line of ^208^Tl and the 338.4, 911 and 968.9-keV γ-ray lines of ^228^Ac. To determine the activity concentration of ^40^K, the 1460.8-keV γ-ray line of this isotope was used. In addition, counts of the 661.66 keV γ-ray region of ^137^Cs were taken to assess the ^137^Cs contamination [6–8].

## RESULTS AND DISCUSSION

The activity concentrations of ^238^U, ^232^Th, ^40^K and ^137^Cs radionuclides in the soil from five locations at the Oodalia Tea Estate in the Fatickchari region of the Chittagong district, Bangladesh (at depths of 0–5, 6–12 and 13–20 cm) are shown in Table 2. The mean activity of ^238^U ranged from 34 ± 9 to 45 ± 3 Bq kg^−1^, ^232^Th ranged from 50 ± 13 to 65 ± 21 Bq kg^−1^ and ^40^K ranged from 245 ± 30 to 635 ± 35 Bq kg^−1^. In topsoil from up to 5-cm depth, ^137^Cs was detected within the range of 3–10 Bq kg^−1^. The mean activity concentrations of ^232^Th, ^238^U, ^40^K and ^137^Cs (in Bq kg^−1^) as found in the present study are compared in Table 3 with other values reported from near the study area [9].

**Table 2.** The activity concentrations of radionuclides in the soil of the Oodalia Tea Estate in the Fatickchari region of Chittagong district of Bangladesh

Radionuclide	Activity concentrations (Bq kg^−1^)					
	Depth cm	Location 1	Location 2	Location 3	Location 4	Location 5
^238^U	0–5	43 ± 8	32 ± 8	45 ± 8	36 ± 7	29 ± 7
	6–12	43 ± 8	27 ± 7	34 ± 7	43 ± 8	43 ± 8
	13–20	48 ± 8	44 ± 8	33 ± 8	30 ± 8	53 ± 8
	**Mean**	**45 ± 3**	**34 ± 9**	**37 ± 7**	**36 ± 7**	**42 ± 12**
^232^Th	0–5	51 ± 11	66 ± 11	62 ± 11	42 ± 11	29 ± 7
	6–12	52 ± 11	66 ± 8	53 ± 11	82 ± 11	55 ± 11
	13–20	51 ± 11	58 ± 11	36 ± 11	72 ± 11	66 ± 11
	**Mean**	**51 ± 1**	**63 ± 5**	**50 ± 13**	**65 ± 21**	**50 ± 19**
^40^K	0–5	264 ± 77	603 ± 79	362 ± 78	342 ± 77	211 ± 76
	6–12	201 ± 78	672 ± 81	474 ± 79	320 ± 78	268 ± 78
	13–20	359 ± 79	630 ± 80	336 ± 79	456 ± 79	256 ± 78
	**Mean**	**275 ± 80**	**635 ± 35**	**391 ± 73**	**373 ± 73**	**245 ± 30**
^137^Cs	0–5	10 ± 1	7 ± 1	9 ± 1	4 ± 1	3 ± 1
	6–12	7 ± 1	ND	ND	ND	ND
	13–20	ND	ND	ND	ND	ND

ND = not detectable.

**Table 3.** Activity concentrations (Bq.Kg^−1^) ^232^Th, ^238^U, ^40^K of soil samples of present study area and nearby area [9] of Chittagong.

SL no.	Name of location	Geographical position		^232^Th Bq kg^−1^	^238^U Bq kg^−1^	^40^K Bq kg^−1^	^137^Cs Bq kg^−1^
		Latitude	Longitude				
1	Panchlaish	22°21′36.89"N	91°50′15.50"E	38.56 ± 2.32	17.06 ± 3.90	455.57 ± 43.99	
2	Baizid Bostami	22°23′18.31"N	91°48′33.49"E	44.08 ± 4.03	24.50 ± 5.43	412.13 ± 36.17	
3	Kulshi	22°21′20.91"N	91°48′42.91"E	43.43 ± 2.44	23.45 ± 4.30	409.08 ± 35.86	
4	Pahartali	22°21′56.84"N	91°46′41.62"E	28.81 ± 2.21	18.12 ± 3.81	561.16 ± 54.21	
5	Kotowali	22°20′29.09"N	91°49′37.05"E	65.87 ± 3.02	31.93 ± 5.11	589.91 ± 66.44	
6	Double Mooring	22°19′46.97"N	91°48′26.85"E	38.10 ± 2.17	20.18 ± 3.75	499.06 ± 37.32	
7	Parhartali	22°21′56.84"N	91°46′41.62"E	31.12 ± 2.27	18.26 ± 3.90	387.06 ± 42.56	
8	Hathazari	22°30′30.47"N	91°48′26.85"E	35.38 ± 2.62	17.29 ± 4.59	495.09 ± 48.66	
9	Chittagong University area	22°28′11.73"N	91°47′25.45"E	32.05 ± 2.43	21.25 ± 4.65	412.48 ± 34.72	
10	Present study	22°36′15.57"N	91°45′18.23"E	50 ± 13 to 65 ± 21	34 ± 9 to 45 ± 3	245 ± 30 to 635 ± 35	3–10

Activity concentrations of ^238^U and ^232^Th in the Oodalia Tea Estate soil were found to be higher than those of the nearby areas, but the activity concentration of ^40^K was found to be lower, as shown in Table 3. An activity concentration of ^137^Cs was found in the present study but not in the other study areas. It appears that the natural soil of the hilly region is radiologically different from that of the nearby regions. The derived outdoor and indoor absorbed dose rates are indicated in Table 4. The outdoor absorbed dose rate in air at 1 m above the ground surface was calculated using the conversion factors given in the UNSCEAR 1988 report [1]:

(1) $${\text{D}}_{\mathrm{outdoor}}=(0.{\text{427 C}}_{U}+0.{\text{662 C}}_{\mathrm{Th}}+0.0{\text{43 C}}_{K}){\text{nGyh}}^{-1},$$

where C_U_, C_Th_ and C_K_ are the average activity concentrations of ^238^U, ^232^Th and ^40^K, respectively, in the samples. The observed outdoor absorbed dose rate was within the range of 52–87 nGy h^−1^, and the mean dose rate of the area was calculated to be 71 nGy h^−1^ (compared with the world average value of 55 nGy h^−1^). The indoor contribution is assumed to be 1.3 times higher than the outdoor dose:

(2) $${\text{D}}_{\mathrm{indoor}}={\text{D}}_{\mathrm{outdoor}}\times \text{1}.\text{3 }({\text{nGy h}}^{-1})[\text{1}].$$

The annual effective dose equivalent from outdoor terrestrial gamma radiation is:

(3) $${\text{D}}_{\mathrm{eff}}=\text{Outdoor dose }({\text{nGy h}}^{-1})\times 0.\text{7 }({\text{Sv Gy}}^{-1})\times \text{876}0({\text{h y}}^{-1})\times 0.\text{2 }\;[\text{1}],$$

where 0.2 is the outdoor occupancy factor and 0.7 Sv Gy^−1^ is the quotient of effective dose equivalent rate over observed dose rate in air (taken from the UNSCEAR Report for environmental exposure to gamma-rays of moderate energy). This value is assumed to apply equally to males and females and to the indoor and outdoor environments. For indoor exposure, using an occupancy factor of 0.8, the annual effective dose equivalent is:

**Table 4.** Outdoor and indoor absorbed dose derived from activity of soil samples

Sample code	Outdoor absorbed dose (nGy h^−1^)	Indoor absorbed dose (nGy h^−1^)	Indoor annual effective dose equivalent (mSv year^−1^)	Outdoor annual effective dose equivalent (mSv year^−1^)	Total annual effective dose equivalent (mSv year^−1^)
HLP1-01	64 ± 14	83 ± 18	0.37 ± 0.08	0.08 ± 0.02	0.45 ± 0.10
HLP1-02	61 ± 14	79 ± 18	0.36 ± 0.08	0.07 ± 0.02	0.43 ± 0.10
HLP1-03	70 ± 14	90 ± 18	0.41 ± 0.08	0.09 ± 0.02	0.50 ± 0.10
HLP2-04	84 ± 14	109 ± 18	0.49 ± 0.08	0.10 ± 0.02	0.59 ± 0.10
HLP2-05	84 ± 12	110 ± 16	0.50 ± 0.07	0.10 ± 0.01	0.60 ± 0.08
HLP2-06	85 ± 14	110 ± 18	0.50 ± 0.08	0.10 ± 0.02	0.60 ± 0.10
NLR1-07	76 ± 14	98 ± 18	0.44 ± 0.08	0.09 ± 0.02	0.53 ± 0.10
NLR1-08	70 ± 14	91 ± 18	0.41 ± 0.08	0.09 ± 0.02	0.50 ± 0.10
NLR1-09	52 ± 14	68 ± 18	0.31 ± 0.08	0.06 ± 0.02	0.37 ± 0.10
NLR2-10	59 ± 14	77 ± 18	0.35 ± 0.08	0.07 ± 0.02	0.42 ± 0.10
NLR2-11	87 ± 14	113 ± 18	0.51 ± 0.08	0.11 ± 0.02	0.62 ± 0.10
NLR2-12	80 ± 14	105 ± 18	0.47 ± 0.08	0.10 ± 0.02	0.57 ± 0.10
KPR-13	55 ± 14	72 ± 18	0.32 ± 0.08	0.07 ± 0.02	0.39 ± 0.10
KPR-14	66 ± 14	86 ± 18	0.39 ± 0.08	0.08 ± 0.02	0.47 ± 0.10
KPR-15	74 ± 14	97 ± 18	0.44 ± 0.08	0.09 ± 0.02	0.53 ± 0.10
**Average**	**71** ± **11**	**92** ± **18**	**0.42** ± **0.07**	**0.09** ± **0.01**	**0.51** ± **0.08**

D_eff_ = indoor dose (nGy h^−1^) × 0.7 (Sv Gy^−1^) × 8760 (h y^−1^) × 0.8.

(4) $${\text{D}}_{\mathrm{eff}}=\text{indoor dose }({\text{nGy h}}^{-1})\times 0.\text{7 }({\text{Sv Gy}}^{-1})\times \text{876}0({\text{h y}}^{-1})\times 0.\text{8}.$$

The results for outdoor, indoor and total annual effective dose equivalents are indicated in Table 4. The average total (outdoor plus indoor) annual effective dose equivalent from terrestrial radiation was found to be 0.51 mSv, of which 0.42 mSv comes from indoor and 0.09 mSv from outdoor; the corresponding world average value is 0.41 mSv, of which 0.34 mSv comes from indoor and 0.07 mSv from outdoor [1].

The parameters external radiation hazard, H_ext_, and internal radiation hazard, H_int_, were calculated using the criterion formulae as follows [10]:

(5) $${\text{H}}_{\mathrm{ext}}={\text{C}}_{U}/\text{37}0+{\text{C}}_{\mathrm{Th}}/\text{259}+{\text{C}}_{K}/\text{481}0\;({\text{Bq kg}}^{-1})\text{ and}$$

(6) $${\text{H}}_{\mathrm{int}}={\text{C}}_{U}/\text{185}+{\text{C}}_{\mathrm{Th}}/\text{259}+{\text{C}}_{K}/\text{481}0\;({\text{Bq kg}}^{-1}).$$

The results for the external and internal radiation hazards are indicated in Table 5. In soil from these locations, both the hazard indices were < 1; the recommended hazard parameters H_ext_ and H_int_ should also be <1.

**Table 5.** External and internal radiation hazard and other radiological parameters derived from activity of soil samples

Sample code	External radiation hazard, H_ext_	Internal radiation hazard, I_int_	Radium equivalent activity, Ra_eq_ (Bq kg^−1^)	Representative level index, I_γr_ (Bq kg^−1^)
HLP1-01	0.37 ± 0.08	0.48 ± 0.10	136 ± 29	0.97 ± 0.24
HLP1-02	0.36 ± 0.08	0.47 ± 0.10	132 ± 29	0.94 ± 0.24
HLP1-03	0.40 ± 0.08	0.53 ± 0.10	148 ± 30	1.07 ± 0.24
HLP2-04	0.47 ± 0.08	0.55 ± 0.10	173 ± 29	1.28 ± 0.24
HLP2-05	0.47 ± 0.07	0.54 ± 0.09	174 ± 25	1.29 ± 0.21
HLP2-06	0.47 ± 0.08	0.59 ± 0.10	176 ± 30	1.29 ± 0.25
NLR1-07	0.43 ± 0.08	0.55 ± 0.10	161 ± 29	1.16 ± 0.24
NLR1-08	0.40 ± 0.08	0.49 ± 0.10	147 ± 29	1.07 ± 0.24
NLR1-09	0.30 ± 0.08	0.39 ± 0.10	110 ± 30	0.80 ± 0.24
NLR2-10	0.34 ± 0.08	0.44 ± 0.10	126 ± 29	0.89 ± 0.23
NLR2-11	0.50 ± 0.08	0.62 ± 0.10	185 ± 30	1.32 ± 0.24
NLR2-12	0.46 ± 0.08	0.54 ± 0.10	169 ± 30	1.22 ± 0.24
KPR-13	0.32 ± 0.08	0.40 ± 0.10	118 ± 29	0.62 ± 0.23
KPR-14	0.38 ± 0.08	0.50 ± 0.10	142 ± 29	1.02 ± 0.24
KPR-15	0.43 ± 0.08	0.57 ± 0.10	160 ± 30	1.18 ± 0.25
Average	0.41 ± 0.06	0.51 ± 0.07	150 ± 23	1.08 ± 0.20

Values for additional radiation hazard parameters—radium equivalent activity, Ra_eq_, and representative level index, I_γr_— were calculated using the formulae:

(7) $${\text{Ra}}_{\mathrm{eq}}=({\text{C}}_{U}+\text{1}.{\text{43C}}_{\mathrm{Th}}+0.0{\text{77C}}_{K})\text{Bq}.{\text{kg}}^{-1}\text{and}$$

(8) $${\text{I}}_{\gamma \;r}=({\text{C}}_{U}/\text{15}0+{\text{C}}_{\mathrm{Th}}/\text{1}00+{\text{C}}_{K}/\text{15}00)\text{Bq}.{\text{kg}}^{-1}[\text{9}].$$

Equation 7 is based on the estimate that 1 Bq kg^−1^ of ^238^U, 0.7 Bq kg^−1^ of ^232^Th or 13 Bq kg^−1^ of ^40^K generate the same γ-ray dose rate. I_γr_ was calculated to indicate different levels of external γ-radiation resulting from different combinations of specific natural activities in specific other materials. This index can be used to estimate the level of γ radiation hazard associated with the natural radionuclides in the materials.

Based on the annual external dose of 1.5 mGy, the safe limits activity in terms of Ra_eq_ is 370 Bq kg^−1^ and in terms of I_γr_ is 1 Bq kg^−1^. It was observed that the Ra_eq_ activity was far below the allowable limit for all the soil samples, and the I_γr_ value was ∼1, as indicated in Table 5. In addition to the natural radionuclides ^137^Cs, a fallout radionuclide, was detected in the topsoil in the range of 3–10 Bq kg^−1^.

## FUNDING

Sample collections were done by Self finance and Radioactivity Testing and Monitoring Laboratory (RTML), Bangladesh Atomic Energy Commission (BAEC) had given opportunity to use the Laboratory for sample preparation and gamma measurements. Open Access publication charges for this article automatically waived as a group A development country author.
